# Letter from the Editor-in-Chief

**DOI:** 10.19102/icrm.2017.080802

**Published:** 2017-08-15

**Authors:** Moussa Mansour


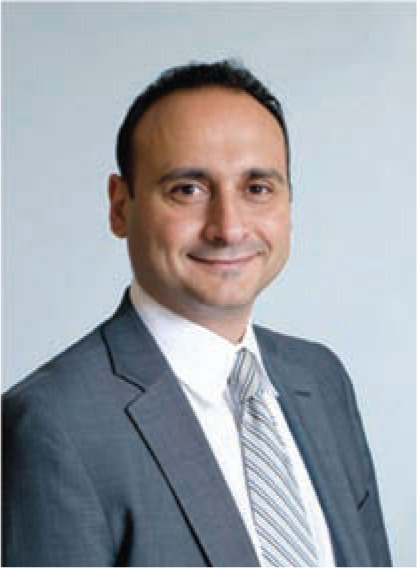


Dear Readers,

The use of electronic health records (EHRs) or electronic medical records (EMRs) has become the standard method for maintaining patients’ medical information in hospitals across the United States and in many parts of the world. They allow for the storage of large amounts of data in an organized and digitalized fashion. Because of them, medical information can be accessed and shared easily across large medical networks. EHRs also allow for the creation of large databases that can assist researchers in considering therapeutic interventions, and in tracking the outcome of treatments implemented in large populations. In addition, they significantly improve the accuracy of medical billing.

One area in cardiology that can benefit most from the use of EHRs is atrial fibrillation (AF) management. This disease affects millions of people in the US, and is the leading cause of cardiogenic stroke. Because of the aging US population and the increased incidence of obesity, hypertension, and sleep apnea, it is expected that the prevalence of AF will continue to increase. Despite numerous studies showing the benefits of oral anticoagulants in patients with AF, the use of these medications remains suboptimal. Population health studies have repeatedly shown that more than 40% of patients with AF and a CHADS score of >2 or more are not prescribed anticoagulation. EHRs are in the unique position to assist with identifying patients with AF who are not treated with oral anticoagulants and helping to design interventions aimed at the initiation of treatment in this group of patients. The detailed information stored in the electronic records can enable the targeting of patients in a personalized manner, in order to reduce the adverse events that may result from these treatments. In addition, EHRs provide patients with have direct access to their medical records and subsequently improve shared decision-making processes between patients and their physicians.

This concept is nicely demonstrated in the article titled “The Use of Electronic Personal Health Records to Improve Medication Adherence and Patient Engagement: A Randomized Study of Non-valvular Atrial Fibrillation Patients” by Chen et al. published in this issue of *The Journal of Innovations in Cardiac Rhythm Management.* Via a randomized study, the authors examined the impact of anticoagulation-specific education delivered via a personal health record on medication adherence. Patients in the intervention arm were allowed to view online, download, and transmit health information. They also received EHR training and medication education via the personal health record, resulting in improved adherence to their medication schedules as compared with the control group.

Notably, this study enrolled only a small number of patients; however it remains important because it highlights two importance points: first, EHRs can be used to implement and track interventions aimed at improving health care delivery; and second, EHRs allow shared decision-making to occur by designating the patient as an integral part of the treatment plan. Larger studies are needed to prove the benefits of such interventions on more critical endpoints, such as survival and stroke.

I hope that enjoy reading this issue of the Journal.

Sincerely,


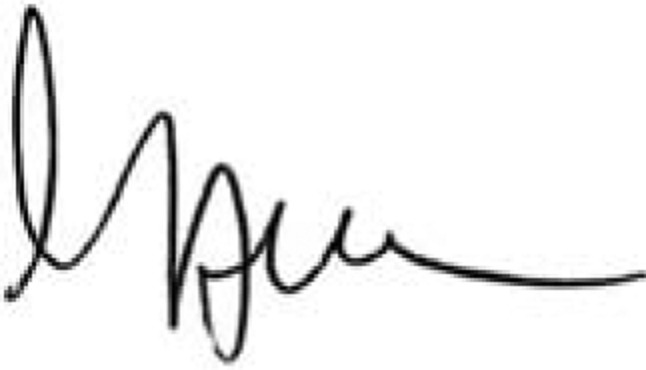


Moussa Mansour, MD, FHRS, FACC

Editor-in-Chief

The Journal of Innovations in Cardiac Rhythm Management

MMansour@InnovationsInCRM.com

Director, Atrial Fibrillation Program

Massachusetts General Hospital

Boston, MA 02114

